# Structural Design and Kinematic Modeling of Highly Biomimetic Flapping-Wing Aircraft with Perching Functionality

**DOI:** 10.3390/biomimetics9120736

**Published:** 2024-12-03

**Authors:** Wenyang Pu, Qiang Shen, Yuhang Yang, Yiming Lu, Yaojie Yan

**Affiliations:** 1 School of Mechatronical Engineering, Beijing Institute of Technology, Beijing 100081, China; bit82shen@bit.edu.cn (Q.S.); 1120213313@bit.edu.cn (Y.Y.); 1120212037@bit.edu.cn (Y.L.); 1120211199@bit.edu.cn (Y.Y.); 2 Beijing Institute of Technology Chongqing Innovation Center, Chongqing 401135, China

**Keywords:** highly biomimetic, flapping-wing aircraft, perching function, kinematics model

## Abstract

Birds use their claws to perch on branches, which helps them to recover energy and observe their surroundings; however, most biomimetic flapping-wing aircraft can only fly, not perch. This study was conducted on the basis of bionic principles to replicate birds’ claw and wing movements in order to design a highly biomimetic flapping-wing aircraft capable of perching. First, a posture conversion module with a multi-motor hemispherical gear structure allows the aircraft to flap, twist, swing, and transition between its folded and unfolded states. The perching module, based on helical motion, converts the motor’s rotational movement into axial movement to extend and retract the claws, enabling the aircraft to perch. The head and tail motion module has a dual motor that enables the aircraft’s head and tail to move as flexibly as a bird’s. Kinematic models of the main functional modules are established and verified for accuracy. Functional experiments on the prototype show that it can perform all perching actions, demonstrating multi-modal motion capabilities and providing a foundation upon which to develop dynamics models and control methods for highly biomimetic flapping-wing aircraft with perching functionality.

## 1. Introduction

Flight is an energy-consuming process, and for most birds in nature, continuous flying significantly depletes their energy reserves; therefore, perching is essential, as it allows birds to recover strength, observe their surroundings, monitor their territory, and search for food [1,2]. Similarly, equipping flapping-wing aircraft with perching capabilities can expand their market potential. During missions, these aircraft can conserve energy by perching on objects such as power poles or branches, thereby extending their endurance and mission duration [3,4].

Although perching technology has significant application potential, achieving perching capabilities in flapping-wing aircraft is challenging. Firstly, in terms of structural design, birds perch not solely through the action of their claws but also through the coordinated movement of their wings, body, and claws [5]. Therefore, for a flapping-wing aircraft to have perching functionality, not only a grasping mechanism similar to bird claws but also multi-degree-of-freedom movement and multi-modal flight capabilities are required, which would allow the aircraft to mimic the wing movements of birds during perching, fully utilize aerodynamic forces, and reduce the probability of perching failure [6,7].

Additionally, compared to fixed-wing aircraft, flapping-wing flight involves low-Reynolds-number unsteady aerodynamics, making high-precision modeling of flapping motion difficult and leading to lower control accuracy, which increases the difficulty of achieving perching [8]. Aside from a few small birds, such as hummingbirds, most birds cannot hover [9]; therefore, to ensure successful perching, it is necessary to minimize forward flight speed and adjust flight posture during perching, which requires coordinated operation of the drive, flapping, and perching mechanisms, thus demanding higher precision in positioning and recognition capabilities, high-impact material resistance, and precise motor speed control for the flapping-wing aircraft.

Currently, there are various solutions for enabling aircraft to perch on resting surfaces, with widely used methods including biomimetic micro-spines, spines, fiber-based adhesives, and nature-inspired mechanical grippers [10,11,12,13,14,15]; these solutions have been applied to fixed-wing aircraft, rotary-wing aircraft, and flapping-wing aircraft. For example, Desbiens et al. [10] designed a perching mechanism for fixed-wing aircraft based on high-friction origami spines, enabling the aircraft to attach to branches; Zhang et al. [16] developed a perching device for rotary-wing aircraft using a bistable clamping mechanism, allowing the aircraft to hang from wall protrusions; and Roderick et al. [17] proposed a bird-inspired perching mechanism for rotary-wing aircraft, featuring a multi-joint design that requires precise control of the locking mechanism to achieve strong grasping capabilities.

In the field of flapping-wing aircraft, Graule et al. [18] conducted research on rapid perching techniques for micro flapping-wing aircraft using Robobee, developed by Harvard University, an approach that employs switchable electrostatic adhesion technology, enabling Robobee to quickly perch on various surfaces, including wood, glass, and natural leaves. Experimental results showed that this technology allows for repeatable transitions from flight to perching, providing stable attachment to different materials. Pakpong et al. [19] designed an iterative learning controller for Robobee, using a model-free error estimation algorithm to improve landing accuracy, thereby enhancing attitude control under high-maneuverability conditions, enabling perching on vertical walls.

Zhao et al. [20] successfully designed a perching micro flapping-wing aircraft by installing a spike at the tail, allowing it to perch on surfaces such as walls and tree trunks, although it requires a certain hardness of the perching surface. Zufferey et al. [21] developed a bird claw mechanism for a 700 g class flapping-wing aircraft to achieve perching on branches. Experimental data demonstrated that the biomimetic bird claw could lock onto objects within 25 ms, meeting the maneuverability and response speed requirements for perching. During perching, releasing the bird claw’s legs can increase the initial speed of the flapping-wing aircraft to 2.5 m/s, enabling re-flight; however, the perching flapping-wing aircraft designed by Zufferey et al. [21] has limited degrees of freedom in its wing movements, preventing full utilization of aerodynamic forces. Additionally, the aircraft lacks a highly biomimetic appearance, and its wings cannot be retracted after perching, resulting in a lack of camouflage and concealment capabilities.

In summary, few medium and large flapping-wing aircraft possess highly biomimetic appearances while also exhibiting bird-like wing flexibility and perching capability. Therefore, we apply bionic principles to achieve the functionality of bird claws and multi-degree-of-freedom wing movements through mechanical structures, designing a highly biomimetic flapping-wing aircraft with perching capabilities. Using a multi-motor scheme and a hemispherical gear structure, we designed a posture conversion module that allows the wings to achieve flapping, twisting, and swinging motions, as well as transition between folded and unfolded states, thus enhancing biomimicry and improving aerodynamic efficiency. A perching module was designed based on the principle of helical motion, converting the motor’s rotational movement into axial translational movement in order to extend and retract the claws, thereby providing the aircraft with perching capability, reducing energy consumption, and increasing mission duration. Utilizing the principles of dual-motor-coupled motion and a limiting structure, we designed a head–tail motion module that allows the head and tail of the biomimetic flapping-wing aircraft to move as flexibly as those of birds.

The main differences and merits of the design method proposed in this paper for a highly biomimetic flapping-wing aircraft with perching capabilities, compared to existing work, are as follows:

(1) Overall design

Existing flapping-wing aircrafts with perching capabilities typically focus on designing a claw-like grasping mechanism similar to bird claws, aiming to minimize the response time of the grasping mechanism and finding an optimal combination of flight speed and response time to achieve perching [17,21,22,23,24]; while this approach often shows good performance in laboratory settings, its applicability in natural environments—where airflow speed and direction change in real time—remains uncertain. In our current proposal, we focused not only on designing a grasping mechanism akin to bird claws but also on addressing the perching process of medium- and large-sized birds. By capturing the postures at various stages of the perching process, the corresponding functional mechanisms were designed to endow the flapping-wing aircraft with bird-like agility and real-time posture adjustment capabilities, thus enabling the aircraft to change its flight posture according to real-time airflow conditions, fully utilizing aerodynamic forces, enhancing disturbance resistance, and reducing energy consumption.

(2) Main functional module design

Posture conversion mechanism: Current medium- and large-sized flapping-wing aircrafts often achieve multi-degree-of-freedom wing movements through combined or hybrid drive methods [25,26,27]; however, these methods involve complex coupled movements between different modes, resulting in high control complexity. The posture conversion mechanism designed in this study adopts a split-drive method to achieve flapping, twisting, and sweeping motions, as well as coupled movements, making it easier to decouple the wing’s motions and reduce the complexity of wing control.

Perching module: Existing flapping-wing aircraft prototypes capable of perching often achieve claw opening and closing through a flexible rope pulled via a slider [21], a design prone to material wear and significant mechanical loss. In this study, we replace the flexible rope and slider with a newly designed opening and closing mechanism, improving mechanical efficiency while addressing material wear issues.

The novelty of our proposal lies in using the primary postures involved in the multi-modal motions of hawks and similar birds as bionic models to design functional modules that enable the flapping-wing aircraft to achieve multi-modal motions such as multi-degree-of-freedom flight and perching, which are not typically found in most biomimetic designs that focus solely on flight. Specifically, we accomplish the following:

(1) We design a posture conversion mechanism that enables the flapping wings to not only perform basic movements such as twisting, flapping, swinging, and folding but also to transition from being retracted against the sides of the body to fully extended, or vice versa, increasing the biomimetic fidelity of the design while also enhancing the flapping-wing aircraft’s multi-modal motion capabilities, in addition to its basic flight abilities.

(2) The development of a dedicated perching module based on helical motion, which allows the claws to extend and retract through axial movement, is a novel mechanism not commonly seen in other flapping-wing aircraft; this mechanism enables the aircraft to replicate the bird-like behavior of perching, which is crucial for energy recovery and environmental observation.

In summary, the novelty in this study lies in combining flight and perching abilities; integrating coordinated wing, claw, head, and tail movements; and developing a functional prototype that demonstrates these capabilities, thus paving the way for more versatile biomimetic flapping-wing aircraft with perching functionality.

## 2. Characteristic Analysis of the Bird Perching Process

### 2.1. Basic Flight and Multi-Modal Movement

**Figure 1:**
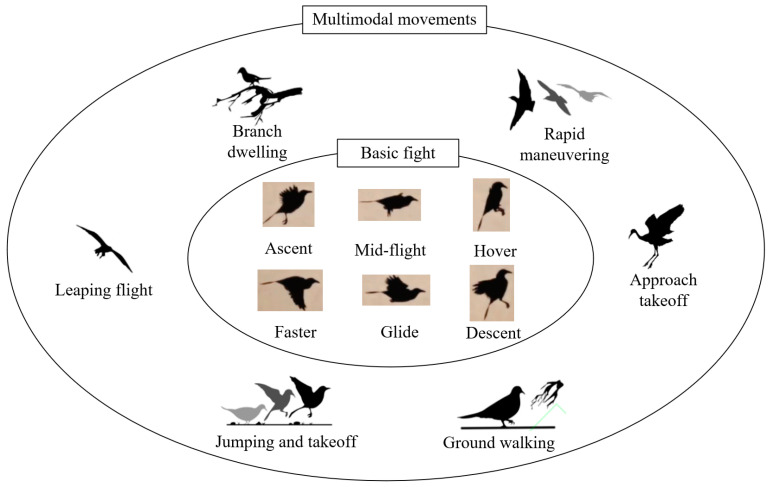
The multi-modal movement patterns of birds.

In addition to fundamental flight postures, such as ascending, level flight, hovering, fast flight, gliding, and descending, birds exhibit multi-modal movements to adapt to their environments; for example, they maintain the ability to take off and land in order to extend their habitat, they utilize intermittent flapping–gliding flight patterns to conserve energy and increase flight duration, and they have developed highly maneuverable flight capabilities that they use to evade predators. The multi-modal movement patterns of birds are illustrated in Figure 1.

### 2.2. Characteristic Analysis

**Figure 2:**
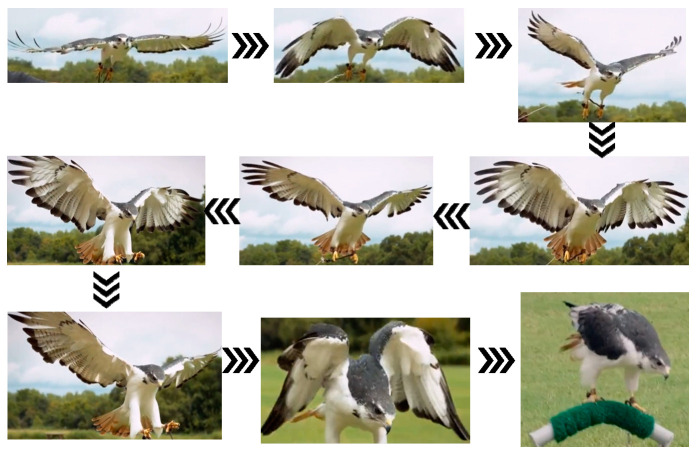
Flight-to-perching posture characteristic diagram.

The perching characteristics of medium and large birds were analyzed using hawks as an example; their perching process is shown in Figure 2, where it can be observed that they first fly rapidly toward the perch with their wings fully extended and continuously flapping. Their legs and claws then stretch backward, parallel to the body axis, maintaining an aerodynamic shape to reduce resistance. As they approach the perch, hawks quickly twist their wings and fully spread their feathers to increase the contact area with the air, thereby reducing their speed. Simultaneously, they decrease the downstroke amplitude of their wings to reduce lift, causing a gradual descent, during which the legs and claws adjust to the proper position, with the claws opening. Upon reaching the perch, the claws quickly tighten and lock onto it, while the wings make fine adjustments to balance thrust and lift, allowing hawks to shift their center of gravity. Once the bird is stable, the wings slowly fold against the sides of the body, completing the perching process.

**Figure 3:**
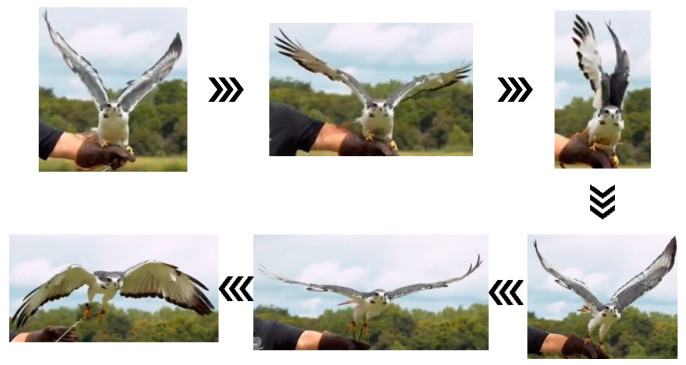
Perching-to-takeoff posture characteristic diagram.

During the initial stage of transitioning from perching to flight, hawks’ wings quickly spread open while their legs bend slightly in preparation for a jump. The body leans forward to maintain a positive angle of attack. As the wings reach their maximum upward angle, the claws extend, and the legs push off to provide initial thrust. Once the claws leave the perch, the legs and claws naturally rotate to align parallel with the body axis, reducing drag. The wings then flap rapidly, folding during the upstroke and fully extending during the downstroke, to increase net lift within each flapping cycle, thereby allowing hawks to achieve flight; the takeoff process of the hawk is shown in Figure 3.

## 3. Structural Design and Functional Analysis

### 3.1. Structural Design

**Figure 4:**
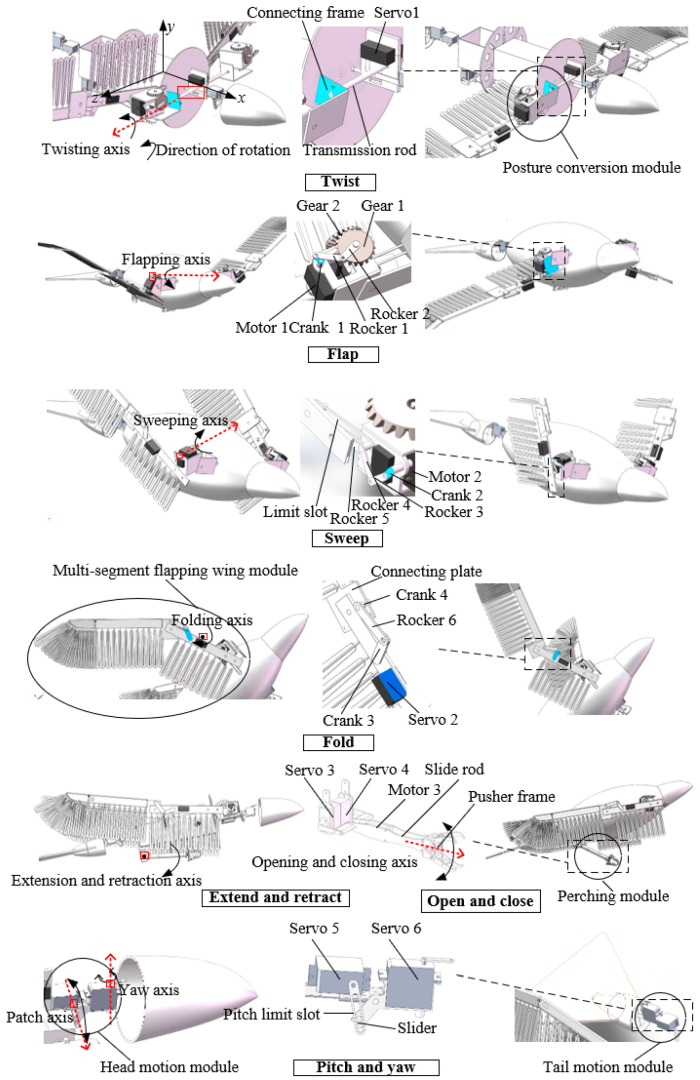
Schematic diagram of the structure and main movement implementation of a flapping-wing aircraft with perching function.

Based on the perching characteristics of the hawk, discussed above, a biomimetic flapping-wing aircraft with perching functionality was designed, as shown in Figure 4. The main functional modules include head motion, tail motion, posture conversion, multi-segment flapping wing, perching, and connection modules.

Using the robotic bird’s center of mass as the origin and the body axis as the *x*-axis, the body coordinate system was established according to the right-hand rule, as shown in Figure 4. Twist is controlled solely via servo 1, which rotates the transmission rod and, consequently, causes the connecting frame to rotate. Since the attitude transformation mechanism is rigidly connected to the linkage, it achieves rotational movement around the *z*-axis, which corresponds to twisting.

Flapping was achieved using motor 1, a crank–rocker mechanism, and gears. Motor 1 serves as the power source for flapping, with its output shaft connected to the rotating shaft. When activated, this connection drives the crank’s rotation, which is transmitted through a two-rocker mechanism, causing gear 1 to swing due to gear meshing. Since gear 2 is fixed, the swinging of gear 1 causes the attitude transformation mechanism to rotate around the *x*-axis to achieve flapping.

Motor 2 and the crank–rocker mechanism work together to achieve a sweeping motion, constrained by the limit slot. The output shaft of motor 2 is connected to the sweeping shaft, and the rotation of the sweeping shaft drives the crank’s rotation, the motion of which is then transmitted through the rocker mechanism, resulting in the sliding of rocker 5 within the limit slot. Since rocker 5 is fixed to the flapping wing, this sliding motion allows the wing to rotate around the *y*-axis, achieving the sweeping motion.

Servo 2 controls the folding motion between the first and second sections of the flapping wing. The output shaft of the servo is connected to the folding axis such that, as the servo rotates, this motion is transmitted through crank 3 to the folding axis. The crank–rocker mechanism then causes rocker 6 to rotate, and the connecting plate, rigidly attached to crank 4, moves in conjunction with rocker 6, enabling the folding motion between the first and second sections of the wing.

Since the movements of the flapping wing are individually controlled by different motors or servos, coordinated control of these mechanisms allows the wing to achieve flapping, twisting, sweeping, folding, and their combinations, thereby enabling the wing to exhibit a level of motion flexibility comparable to that of real birds.

The perching module is driven with servos 3 and 4, as well as motor 3, to perform the corresponding functions. Servos 3 and 4 rotate simultaneously by the same angle to control the extension and retraction of the perching module, mimicking the leg movements of the hawk. Motor 3 drives the sliding rod to rotate synchronously and, under the action of the threaded mechanism, causes the push frame at the bottom of the sliding rod to move translationally, enabling the opening and closing of the perching module’s claws, corresponding to the claw movements of the hawk.

The head and tail motion modules function on the same principle; for example, servo 6 drives the head to perform yaw motion, while the limiting slot converts the rotational movement of servo 5 into the translational motion of the slider, enabling the pitch movement of the head.

The head and tail motion modules independently control the head and tail to achieve pitch, yaw, and dual-channel coupled motions. The head motion not only adjusts the attitude according to flight needs but also enhances task execution capabilities; for example, when combined with a camera, it can increase the monitoring angle. The tail motion provides the necessary yaw, pitch, and roll torques during flight, enabling the aircraft to change flight modes. The detailed introduction of each module follows.

**Figure 5:**
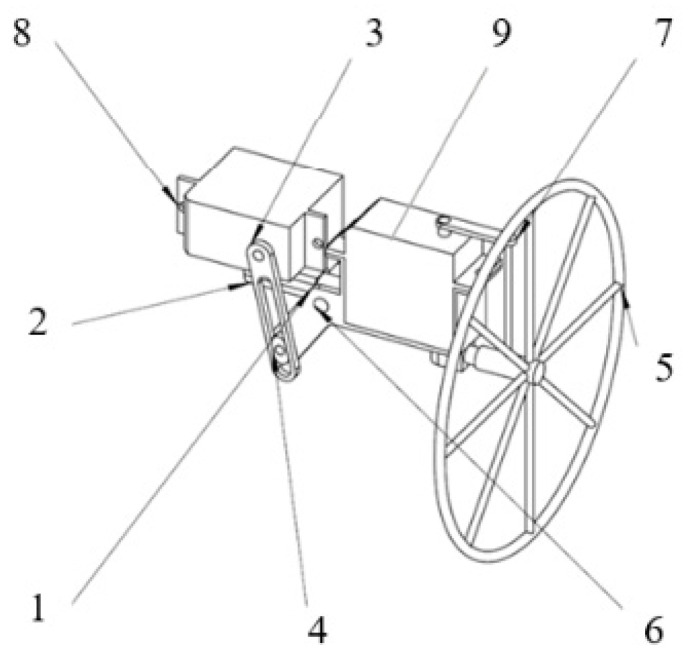
The components of the head motion module (1: pitch swing rocker; 2: fixed sleeve; 3: pitch swing drive rod; 4: slider; 5: yaw swing rocker; 6: connecting pin; 7: yaw swing drive rod; 8: pitch servo; 9: yaw servo).

The head motion module, illustrated in Figure 5, includes a pitch swing rocker, a fixed sleeve, a pitch swing drive rod, a slider, a yaw swing rocker, a connecting pin, a yaw swing drive rod, a pitch servo, and a yaw servo. One end of the fixed sleeve is attached to the frame, while the other end connects to the pitch swing rocker. Both the fixed sleeve and the pitch swing rocker have through-holes, which are joined via the connecting pin to form a hinge. The pitch servo is mounted on the top of the fixed sleeve. The pitch swing drive rod connects the pitch servo to the pitch swing rocker, with its top end attached to the pitch servo’s output shaft and its middle and lower sections featuring a swing slot. The bottom end of the pitch swing rocker has a rotatable protruding shaft, with the slider fitting over this shaft through its own through-hole, thereby allowing it to rotate with the pitch swing rocker. The slider meshes with the swing slot surface of the pitch swing drive rod. The bottom of the yaw servo is fixed to the top of the pitch swing rocker. One end of the yaw swing rocker is attached to the end of the pitch swing rocker farthest from the fixed sleeve, while the other end connects to the aircraft’s head. The yaw swing drive rod connects the output shaft of the yaw servo to the yaw swing rocker. The tail motion module has a structure identical to that of the head motion module.

**Figure 6:**
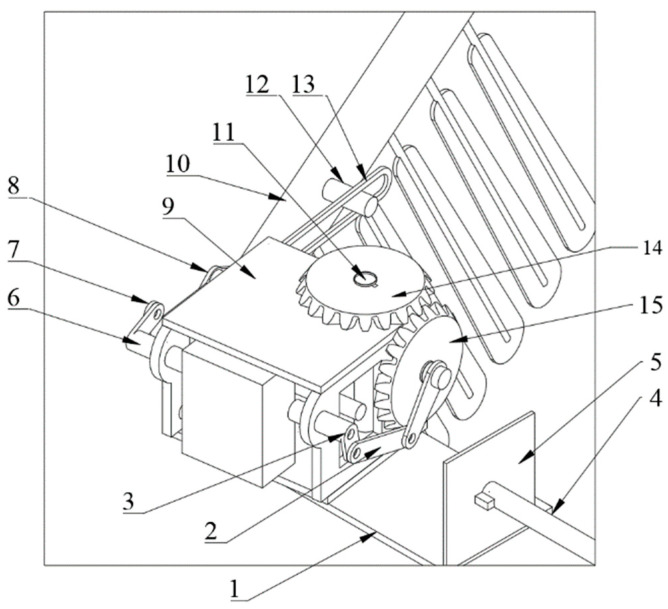
The components of the posture conversion module (1: base; 2: posture conversion link; 3: posture conversion crank; 4: rotational drive rod; 5: connecting plate; 6: folding drive rod; 7: folding link; 8: V-shaped rocker; 9: frame plate; 10: mid-section wing; 11: main shaft; 12: connecting rod; 13: slide rail; 14: gear 1; 15: gear 2).

The posture conversion module allows the wings to perform flapping, twisting, swinging, and coupling movements. A split-drive method is used to achieve multi-degree-of-freedom motion in the flapping wings, with one motor driving the flapping motion and two servos independently driving the twisting and sweeping motions of the wings. As depicted in Figure 6, the posture conversion module includes a base, a frame, a drive motor, a folding servo, a rotational servo, a folding mechanism, a wing folding mechanism, a flapping mechanism, a side plate, a rotational drive rod, and a connecting plate.

The frame is mounted on the base and rotates around a vertical axis. The drive motor and folding servo were installed inside the frame. The drive motor connects to the flapping mechanism, enabling the frame to swing relative to the base and facilitating overall wing flapping. The folding servo is linked to the folding mechanism, allowing the segment of the wing to fold along its span direction. The side plate is positioned on one side of the frame, with its bottom end attached to the base. The rotational drive rod extends horizontally, with one end fixed to the side plate and the other end connected to the rotational servo within the fuselage. The connecting plate, fixed to the fuselage, supports the rotational drive rod. The rotational servo drives the rotational drive rod, enabling the wing to rotate around the axis of the rotational drive rod via the side plate, base, and frame, thereby allowing the wing to transition between extended and folded positions along the sides of the body.

**Figure 7:**
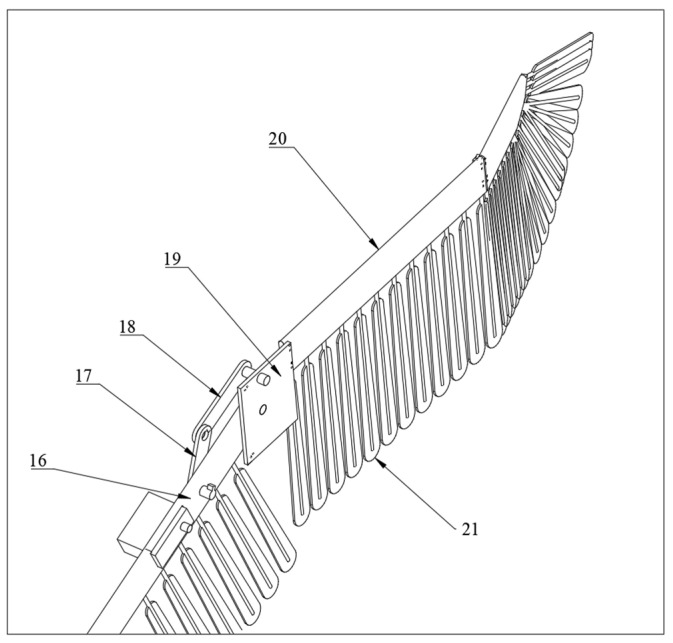
The structure diagram of the multi-segment flapping wing module (16: middle wing segment; 17: folding crank; 18: folding rocker; 19: connecting plate; 20: second outer wing segment; 21: feathers).

The multi-segment flapping wing module generates the aerodynamic forces required for flight and was designed with multiple segments to enhance the range of wing span movements and improve aerodynamic efficiency, with its structure shown in Figure 7. The module includes the first outer wing segment, second outer wing segment, third outer wing segment, a servo, and a folding mechanism. The folding mechanism comprises a folding rocker, a folding crank, and a connecting plate. One end of the folding crank attaches to the servo, while the other end connects to the folding rocker. The other end of the folding rocker is linked to the middle of the connecting plate via a rotating shaft. The wing connecting plate sequentially joins each segment of the flapping wing. The first outer wing segment connects to the second outer wing segment at one end and is secured to the attitude conversion module at the other end. All three outer wing segments are equipped with evenly distributed feathers.

**Figure 8:**
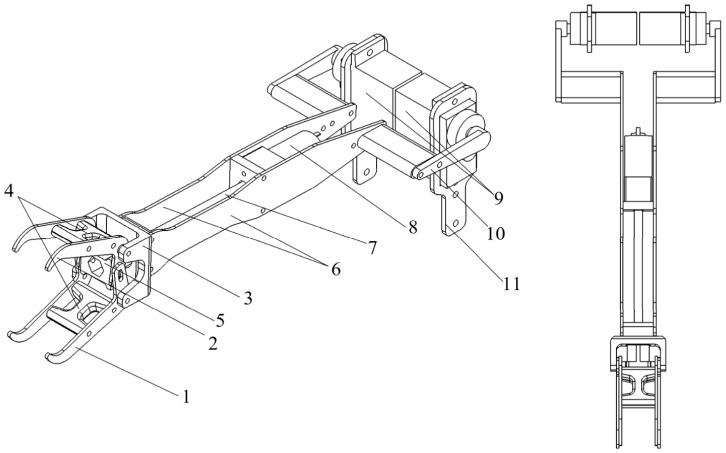
The structure diagram of the perching module (1: lower claw; 2: upper claw; 3: fixed base; 4: claw connector; 5: pusher frame; 6: side plate; 7: slide rod; 8: motor; 9: servo; 10: crank; 11: body connector).

The perching module provides the necessary support force for the aircraft during perching, with its structural components shown in Figure 8. The perching module is primarily composed of claws, a pusher frame, slide rods, side plates, a crank, and body connectors. The body connectors are fixed to the connecting module, and the servo controls the crank’s rotation to retract or deploy the perching module. Both the slide rod and the inner hole of the pusher frame have helical structures. The motor drives the slide rod to rotate, causing the pusher frame to move up and down, thereby opening and closing the claws. Compared to the design method in reference [21], which uses a slider to pull a flexible cable for claw opening and closing, we converted the pusher’s translational movement into claw opening and closing through the claw mechanism design, addressing the issue of material wear in the flexible cable solution. Additionally, the dual-claw design used in this study increases the contact area with the perching object compared to a single claw, enhancing gripping performance.

**Figure 9:**
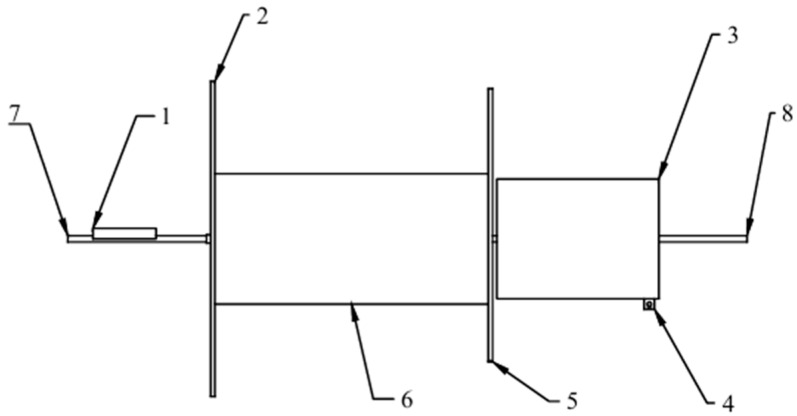
Diagram of the connecting module structure (1: posture conversion module connecting rod; 2: front circular plate; 3: battery fixing plate; 4: perching module connecting rod; 5: rear circular plate; 6: load mounting plate; 7: head connecting rod; 8: tail connecting rod).

The connecting module serves as the supporting framework for the flapping-wing aircraft, as illustrated in Figure 9; it mainly consists of a frame and a streamlined outer shell, structurally connecting the various functional modules and giving the aircraft a bird-like appearance. The frame includes concentrically arranged front and rear circular plates, a posture conversion module connecting rod, a mission payload mounting plate, a battery fixing plate, a head connecting rod, a tail connecting rod, and a perching module connecting rod. The streamlined outer shell is bolted to the front and rear circular plates. The head and tail motion modules are connected to the front and rear ends of the head and tail connecting rods, respectively, through fixed sleeves. The posture conversion module is securely attached to the connecting module via the posture conversion module connecting rod, and the perching module is fixed to the connecting module through the perching module connector.

### 3.2. Functional Analysis

**Figure 10:**
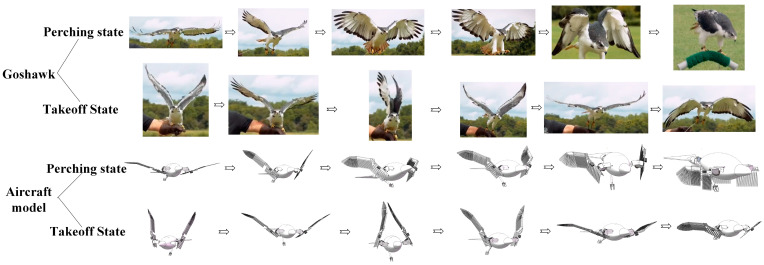
Comparison of the perching and takeoff stages of the flapping-wing aircraft model with the corresponding hawk postures.

Based on the motion process of hawks transitioning from flight to perching (referred to as the perching state) or from perching to taking off (referred to as the takeoff state), we analyzed the functions of the designed perching-capable bionic flapping-wing aircraft. Figure 10 shows the frame-by-frame diagrams of the perching-capable bionic flapping-wing aircraft performing corresponding postures at each stage of the hawk’s perching and takeoff states.

In the perching state, the controller issues commands to adjust the flapping posture and open the claws. The posture conversion module, multi-segment flapping wing module, and perching module execute the controller’s instructions, adjusting the flapping frequency and angle to change the aircraft’s speed and posture. The servo located at the connection between the perching module and the body rotates to adjust the perching module to the appropriate angle. Simultaneously, the motor in the perching module rotates, driving the screw, which causes the push frame to move along the axis of the slide rod, rapidly opening the claws. The motor stops once the claw has fully opened to its maximum angle. The flapping-wing aircraft continuously adjusts its flight posture to maximize aerodynamic efficiency as it approaches the perching point. Upon contact with the perching object (rod-shaped), the mechanical limit device releases the lock, and the claws rapidly tighten under spring force, securing the flapping-wing aircraft to the perching object. The controller then maneuvers the posture conversion module and multi-segment flapping wing module to fold the wings against the sides of the body, completing the perching process.

In the takeoff state, the controller issues commands to adjust the wing posture and open the claw. The posture conversion module, multi-segment flapping wing module, and perching module execute these commands accordingly. The wings quickly unfold from the sides of the body and start flapping at the appropriate angle. Once they reach maximum flapping frequency, the claw opens. Simultaneously, the servo at the connection between the perching module and the body rotates to a preset angle, retracting the perching module into the streamlined outer shell. After the claw disengages from the perch, the mechanical limit device releases the lock, allowing the claw to tighten again, and the aircraft takes off.

## 4. Kinematic Modeling

### 4.1. Posture Conversion Module

**Figure 11:**
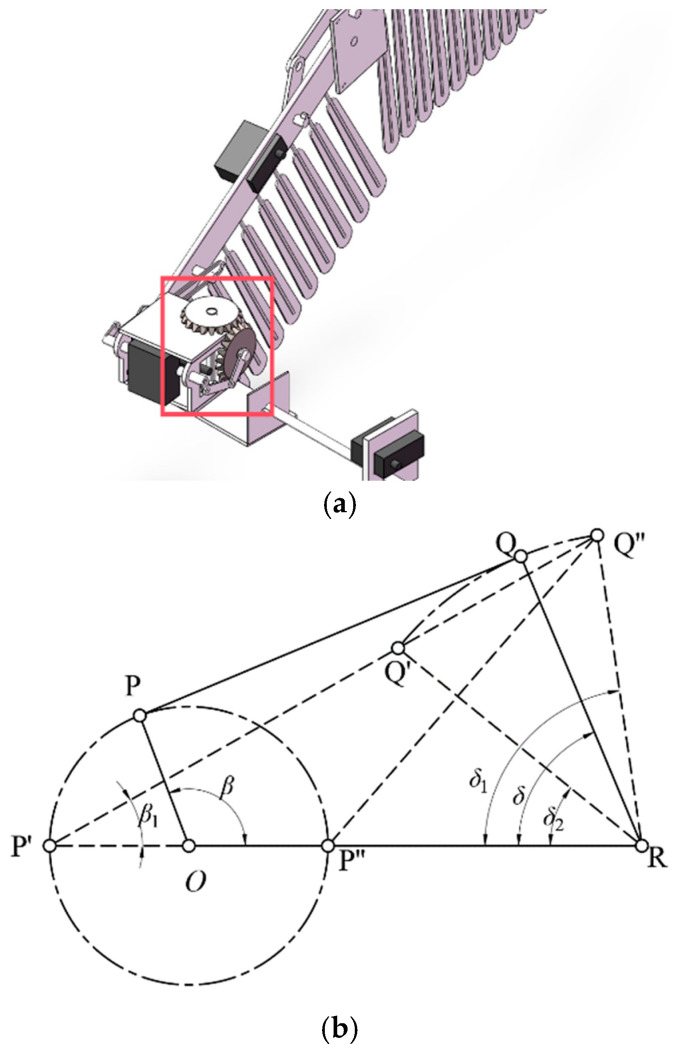
The schematic diagram (**a**) and motion parameters (**b**) of the flapping module. The red box indicates the mechanism corresponding to the motion parameters.

The schematic diagram and motion parameters of the flapping module’s implementation are shown in Figure 11.

As depicted in Figure 6, the flapping angle is related to the dimensions of the flapping mechanism. Taking gear center O as the origin, OP represents the crank, PQ is the connecting rod, QR is the rocker, and RO is the frame. OP rotates around point O, causing QR to reciprocate.

If the crank OP is the driving component and rotates clockwise around point O (0,0), with an angular velocity of *w*_0_ and covering an angle of *w*_0t_, according to mechanical principles, the crank–rocker mechanism will have two extreme motion positions, OP′Q′R (limit one) and OP″Q″R (limit two), as shown in Figure 6.

The maximum flapping angle, denoted as ∠Q′RO, is defined as *δ*_1_; thus, Equation (1), below, can be derived based on geometric relationships.

(1) $$\cos\delta_{1}=\frac{l_{P^{\prime }Q}^{2}-l_{P^{\prime }R}^{2}-l_{Q^{\prime }R}^{2}}{-2\cdot l_{P^{\prime }R}\cdot l_{Q^{\prime }R}}$$

(2) $$\delta_{1}=\arccos\left(\frac{l_{P^{\prime }Q}^{2}-l_{P^{\prime }R}^{2}-l_{Q^{\prime }R}^{2}}{-2\cdot l_{P^{\prime }R}\cdot l_{Q^{\prime }R}}\right)$$

The minimum flapping angle, denoted as ∠Q″RP″, is defined as *δ*_2_, represented using Equation (4), below.

(3) $$\cos\delta_{2}=\frac{l_{P^{\prime \prime }Q^{\prime \prime }}^{2}-l_{P^{\prime \prime }R}^{2}-l_{Q^{\prime \prime }R}^{2}}{-2\cdot l_{P^{\prime \prime }R}\cdot l_{Q^{\prime \prime }R}}$$

(4) $$\delta_{2}=\arccos\left(\frac{l_{P^{\prime \prime }Q^{\prime \prime }}^{2}-l_{P^{\prime \prime }R}^{2}-l_{Q^{\prime \prime }R}^{2}}{-2\cdot l_{P^{\prime \prime }R}\cdot l_{Q^{\prime \prime }R}}\right)$$

To validify the above relationship, it is necessary to satisfy the following:

(5) $$L_{\max}+L_{\min}<L^{\prime }+L^{\prime \prime }$$

where *L*_max_ is the maximum length among OP, PQ, QR, and OR, and *L*_min_ is the minimum length among them. *L*′ and *L*″ denote the lengths of the other links. The flapping angle can be expressed as follows:

(6) $$\delta =\delta_{2}-\delta_{1}=\arccos\left(\frac{l_{P^{\prime \prime }Q^{\prime \prime }}^{2}-l_{P^{\prime \prime }R}^{2}-l_{Q^{\prime \prime }R}^{2}}{-2\cdot l_{P^{\prime \prime }R}\cdot l_{Q^{\prime \prime }R}}\right)-\arccos\left(\frac{l_{P^{\prime }Q}^{2}-l_{P^{\prime }R}^{2}-l_{Q^{\prime }R}^{2}}{-2\cdot l_{P^{\prime }R}\cdot l_{Q^{\prime }R}}\right)$$

### 4.2. Multi-Segment Flapping Wing Module

**Figure 12:**
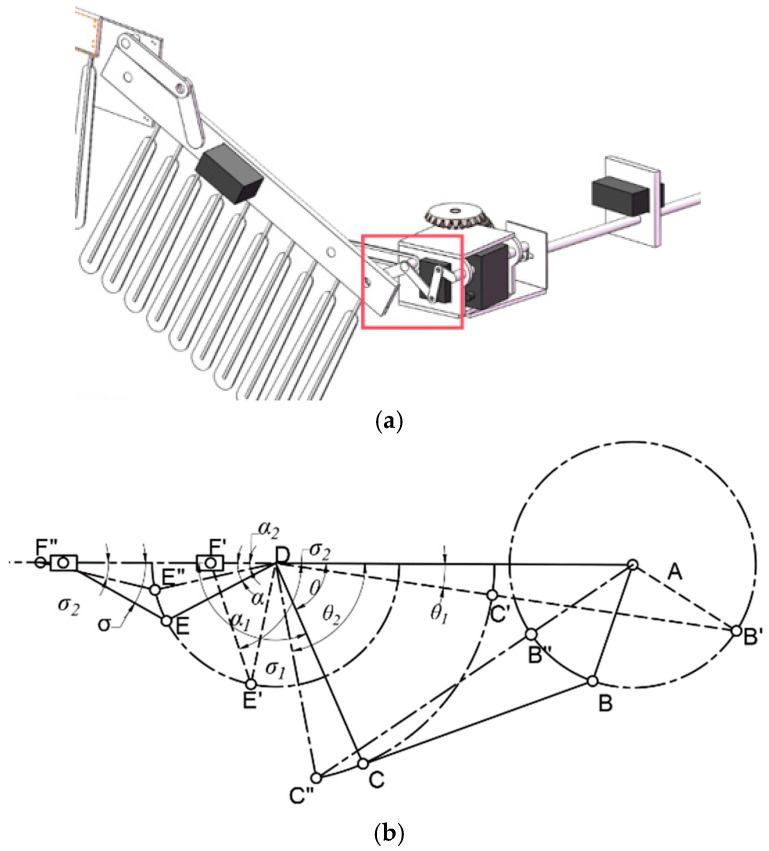
The schematic diagram (**a**) and motion parameters (**b**) of the folding module. The red box indicates the mechanism corresponding to the motion parameters.

The schematic diagram and motion parameters of the folding module implementation are shown in Figure 12, below.

Taking the gear center A as the origin, AB represents the crank, BC is the connecting rod, CD and DE are the rocker arms, and DA represents the frame. When AB rotates around point A, CD reciprocates, where EF is of the same length as DE.

Let the angle between CD and DE be *φ*, and the angle between EF and DF be *σ*. When the rod AB receives the driving force, it rotates clockwise with an angular velocity of *ω*_0_, covering an angle of *ω*_0t_. According to mechanical principles, if the crank AB is the driving component, AB will be collinear with BC twice during one rotation, causing the EF rod to reciprocate along with the CED rod. Due to structural constraints, the crank–rocker mechanism will have two extreme positions, namely ABC′D′ (limit one) and ABC″D″ (limit two), as illustrated in Figure 12.

The maximum folding angle, denoted as ∠C′DA, is *θ*_1_, and ∠F′DE′ is *α*_1_. According to geometric relationships, we can obtain the following equation:

(7) $$\cos\theta_{1}=\frac{l_{AB^{\prime }}^{2}-l_{AD^{\prime }}^{2}-l_{B^{\prime }D}^{2}}{-2\cdot l_{AD}\cdot l_{B^{\prime }D}}$$

(8) $$\theta_{1}=\arccos\left(\cos\delta_{1}=\frac{l_{AB^{\prime }}^{2}-l_{AD^{\prime }}^{2}-l_{B^{\prime }D}^{2}}{-2\cdot l_{AD}\cdot l_{B^{\prime }D}}\right)$$

∠CDE is defined as *φ* and calculated using Equation (9), below.

(9) $$\alpha_{1}=180-\theta_{1}-\varphi$$

The minimum folding angle, denoted as ∠C″DA, is *θ*_2_ and can be solved using Equation (11), below.

(10) $$\cos\theta_{2}=\frac{l_{C^{\prime \prime }A}^{2}-l_{C^{\prime \prime }D}^{2}-l_{AD}^{2}}{-2\cdot l_{C^{\prime \prime }D}\cdot l_{AD}}$$

(11) $$\theta_{2}=\arccos\left(\frac{l_{C^{\prime \prime }A}^{2}-l_{C^{\prime \prime }D}^{2}-l_{AD}^{2}}{-2\cdot l_{C^{\prime \prime }D}\cdot l_{AD}}\right)$$

It is evident that the following relationships exist:

(12) $$\alpha_{2}=180-\theta_{2}-\varphi$$

To satisfy the above relationships, the following conditions must be met:

(13) $$L_{\max}+L_{\min}<L^{\prime }+L^{\prime \prime }$$

where *L*_max_ is the maximum length among AB, BC, CD and AD, and *L*_min_ is the minimum length among them. *L*′ and *L*″ denote the lengths of the other links.

Through the above derivation, we can obtain the folding angle *θ* as follows:

(14) $$\theta =\theta_{2}-\theta_{1}=\arccos\left(\frac{l_{C^{\prime \prime }A}^{2}-l_{C^{\prime \prime }D}^{2}-l_{AD}^{2}}{-2\cdot l_{C^{\prime \prime }D}\cdot l_{AD}}\right)-\arccos\left(\frac{l_{AB^{\prime }}^{2}-l_{AD^{\prime }}^{2}-l_{B^{\prime }D}^{2}}{-2\cdot l_{AD}\cdot l_{B^{\prime }D}}\right)$$

**Figure 13:**
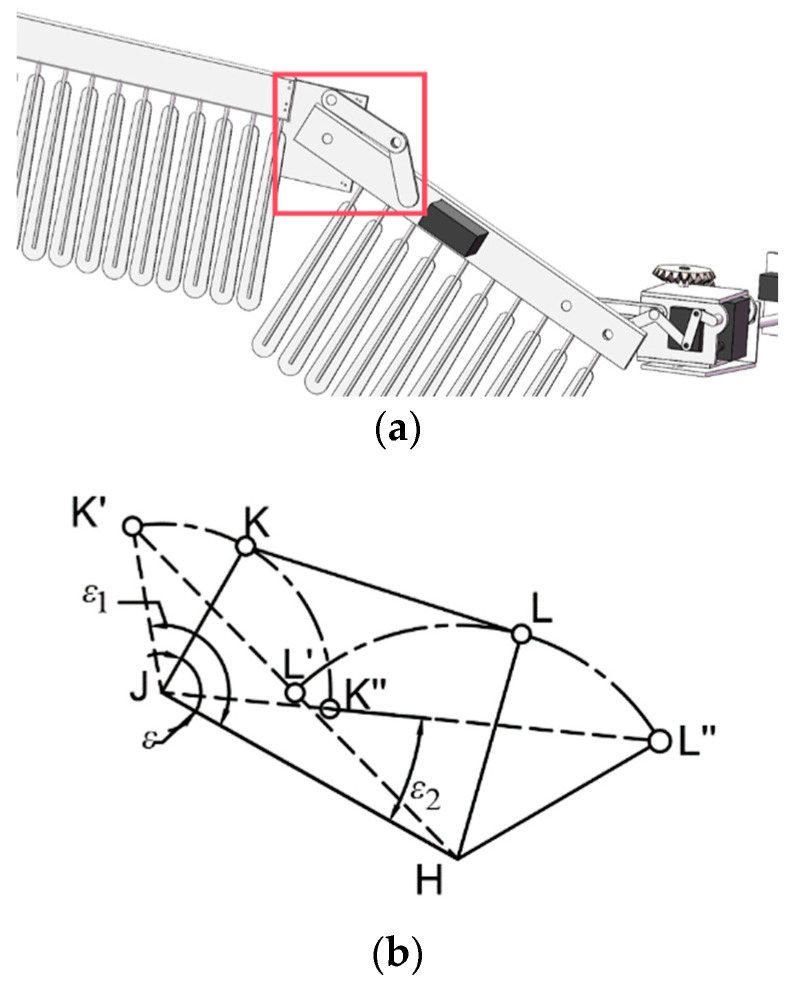
The schematic diagram (**a**) and motion parameters (**b**) of the bending module. The red box indicates the mechanism corresponding to the motion parameters.

The principle diagram and motion parameters of the bending module are shown in Figure 13, below.

As shown in Figure 13, the mechanism formed by JKLH constitutes a double rocker mechanism. JK and LH serve as rockers, limited to swinging within a specific range, rather than completing full rotations. KL acts as a coupler, while JH functions as the frame. The LH rocker drives the motion of the JK rocker, reaching its limit position and then reversing the driving force to achieve reciprocating motion.

The coordinate of the point H, the center of rotation, is (0,0), and the LH rod rotates clockwise upon receiving the driving force, with an angular velocity of ω_0_ and a rotation angle of *ω*_0t_. Due to structural constraints, the crank–rocker mechanism will have two extreme motion positions, JK′L′H (limit one) and JK′L′H (limit two), as illustrated in the figure.

The maximum bending angle, denoted as ∠HJK′, is *ε*_1_, which can be expressed as follows:

(15) $$\cos\varepsilon_{1}=\frac{{\left(l_{K^{\prime }L^{\prime }}+l_{L^{\prime }H}\right)}^{2}-l_{JK^{\prime }}^{2}-l_{JH}^{2}}{-2\cdot l_{JK^{\prime }}\cdot l_{JH}}$$

(16) $$\varepsilon_{1}=\arccos\left(\frac{{\left(l_{K^{\prime }L^{\prime }}+l_{L^{\prime }H}\right)}^{2}-l_{JK^{\prime }}^{2}-l_{JH}^{2}}{-2\cdot l_{JK^{\prime }}\cdot l_{JH}}\right)$$

Similarly, the minimum bending angle of ∠HJK′ is obtained as follows:

(17) $$\cos\varepsilon_{2}=\frac{l_{L^{\prime \prime }H}^{2}-{\left(l_{JK^{\prime \prime }}+l_{K^{\prime \prime }L^{\prime \prime }}\right)}^{2}-l_{JH}^{2}}{-2\cdot (l_{JK^{\prime \prime }}+l_{K^{\prime \prime }L^{\prime \prime }})\cdot l_{JH}}$$

(18) $$\cos\varepsilon_{2}=\arccos\left(\frac{l_{L^{\prime \prime }H}^{2}-{\left(l_{JK^{\prime \prime }}+l_{K^{\prime \prime }L^{\prime \prime }}\right)}^{2}-l_{JH}^{2}}{-2\cdot (l_{JK^{\prime \prime }}+l_{K^{\prime \prime }L^{\prime \prime }})\cdot l_{JH}}\right)$$

To satisfy the above relationships, the following conditions must be met:

(19) $$L_{\max}+L_{\min}<L^{\prime }+L^{\prime \prime }$$

where *L*_max_ is the maximum length among JK, KL, LH and JH, and *L*_min_ is the minimum length among them. *L*′ and *L*″ denote the lengths of the other links.

Through the above derivation, we can obtain the folding angle *ε* as follows:

(20) $$\varepsilon =\varepsilon_{2}-\varepsilon_{1}=\arccos\left(\frac{l_{L^{\prime \prime }H}^{2}-{\left(l_{JK^{\prime \prime }}+l_{K^{\prime \prime }L^{\prime \prime }}\right)}^{2}-l_{JH}^{2}}{-2\cdot (l_{JK^{\prime \prime }}+l_{K^{\prime \prime }L^{\prime \prime }})\cdot l_{JH}}\right)-\arccos\left(\frac{{\left(l_{K^{\prime }L^{\prime }}+l_{L^{\prime }H}\right)}^{2}-l_{JK^{\prime }}^{2}-l_{JH}^{2}}{-2\cdot l_{JK^{\prime }}\cdot l_{JH}}\right)$$

### 4.3. Perching Module

**Figure 14:**
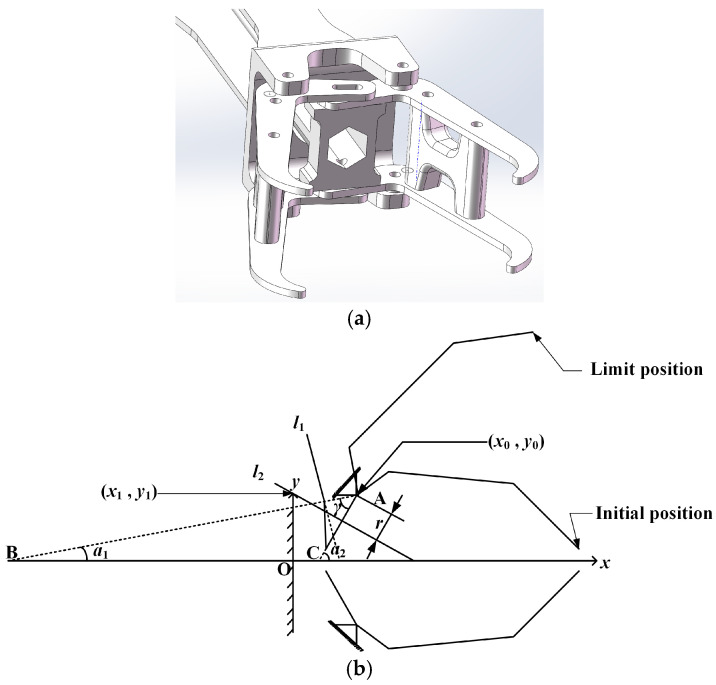
The schematic diagram (**a**) and motion parameters (**b**) of the perching module.

The principle diagram and motion parameters of the perching module are shown in Figure 14, below.

As shown in Figure 14, a Cartesian coordinate system *Oxy* is established. The coordinates of the claw’s rotation center are (*x*_0_, *y*_0_), and the mechanical limit points for the claw’s rotation are (*x*_1_, *y*_1_). The rods *l*_1_ and *l*_2_ represent the mechanical limit positions of the claw at the initial and limit positions, respectively, and their positions determine the opening and closing angle of the claw. Let the slopes of the lines on which *l*_1_ and *l*_2_ lie be *k*_1_ and *k*_2_, respectively. Points A and C are taken on the *x*-axis such that AB is perpendicular to *l*_1_ and AC is perpendicular to *l*_2_. According to the rigid body rotation theorem, the distance from the rotation center (*x*_0_, *y*_0_) to both *l*_1_ and *l*_2_ is *r*. The angle between AB and the positive *x*-axis is *α*_1_, and the angle between AC and the positive *x*-axis is *α*_2_.

The equation of the line where *l*_2_ is located is the following:

(21) $$y-y_{1}=k_{2}(x-x_{1})$$

According to the distance formula from a point to a straight line, the distance from the rotation center to *l*_2_ is as follows:

(22) $$r=\frac{\left|k_{2}x_{0}-y_{0}+y_{1}-k_{2}x_{1}\right|}{\sqrt{1+k_{2}^{2}}}$$

By organizing Equation (22), we can obtain the following:

(23) $$[{\left(x_{0}-x_{1}\right)}^{2}-r^{2}]k_{2}^{2}+2(x_{0}-x_{1})(y_{1}-y_{0})k_{2}+{\left(y_{1}-y_{0}\right)}^{2}-r^{2}=0$$

According to geometric relationships, the opening and closing angle can be expressed as follows:

(24) $$\gamma =\alpha_{1}-\alpha_{2}=\tan^{-1}(1/k_{2})-\tan^{-1}(1/k_{1})$$

### 4.4. Head Motion Module

**Figure 15:**
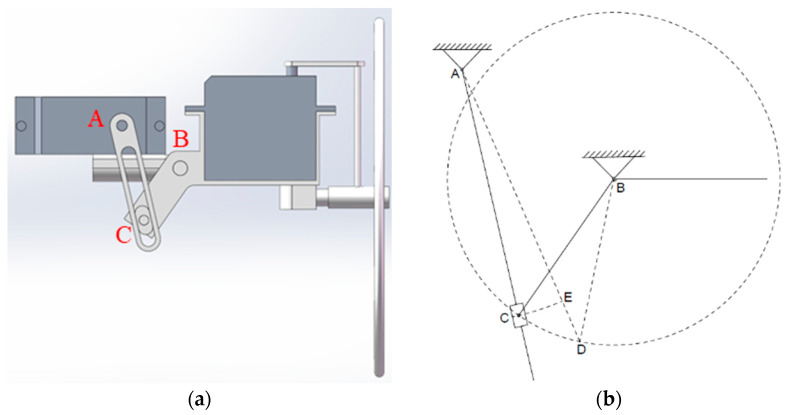
The schematic diagram (**a**) and motion parameters (**b**) of the head motion module. The letters in the schematic diagram (**left**) correspond to the motion parameters (**right**).

Since the principle diagram and mechanism schematic of the head and tail motion modules are identical, this section models only the head motion module. The principle diagram and mechanism schematic of the head motion module are shown in Figure 15, below.

In Figure 15, ACB represents the initial position of the head motion module. In the initial state, both the pitch and yaw angles of the head are 0. Let the initial length of rod AC be *l*_0_, and the change in length during rotation be Δ*l*; the length of rod BC is *R*_0_, and the distance between points A and B is *l*_AB_. According to the cosine theorem, the following is true:

(25) $$\cos\alpha =\frac{l_{AB}^{2}+R_{0}^{2}-{\left(l_{0}+\Delta l\right)}^{2}}{2l_{AB}R_{0}}$$

The pitch angle of the head motion module can be calculated as follows:

(26) $$\theta =\alpha -\theta_{0}=\cos^{-1}\frac{l_{AB}^{2}+R_{0}^{2}-{\left(l_{0}+\Delta l\right)}^{2}}{2l_{AB}R_{0}}-\theta_{0}$$

## 5. Verification of Kinematic Model Accuracy and Prototype Performance Experiment

### 5.1. Kinematic Model Accuracy Simulation

**Figure 16:**
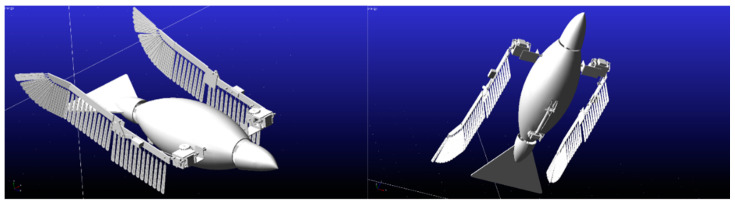
ADAMS model of flapping wing aircraft with perching function.

The key motion parameters of the flapping-wing aircraft were simulated using ADAMS, the imported model for which is shown in Figure 16. In order to resolve the issue in the posture conversion module, where the rotational axes of two revolute joints (gears) are not coplanar and thus cannot mesh, a force-driven contact constraint was added to enable the coupled rotation of the non-coplanar gears.

With target angles of 120° for flapping, 105° for folding, 50° for retracting, 120° for opening and closing, 20° for head and tail pitching, and 40° for head and tail yawing, the specific dimensions of the main component were determined based on the established kinematic model, as shown in Table 1.

**Figure 17:**
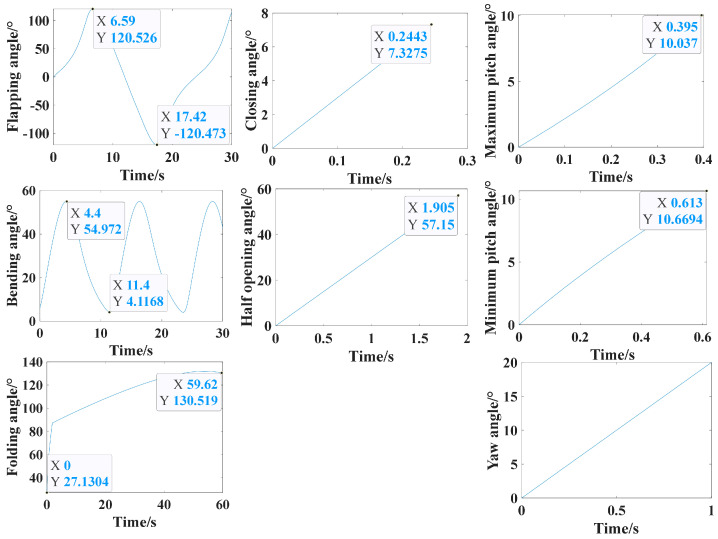
The simulation results of each module.

The simulation results of each module are illustrated in Figure 17. Motion parameters associated with the posture conversion module, including the flapping, folding, and bending angles, are depicted in the first column. The second column shows the results related to the opening and closing angle of the perching module. The pitching and yawing angles of the head coupling motion module are tested and presented in the third column, and these results are also applicable to the tail coupling module.

The comparison between the simulation results and theoretical calculations is shown in Table 2. Deviation is calculated using Equation (27), as follows:

(27) $$Deviation=\frac{v_{cal}-v_{sim}}{v_{sta}}\times 100\%$$

where *v_cal_* represents the calculation value, *v_sim_* is the simulation value, and *v_sta_* represents the standard value.

As shown in Table 2, the calculated values are generally consistent with the simulation results, with the maximum deviation being 6.8% (for the opening and closing angle), a deviation possibly due to the theoretical calculations simplifying the perching module and making assumptions that overlook real-world factors such as friction, deformation, and gaps.

### 5.2. Kinematic Model Accuracy Experiment

**Figure 18:**
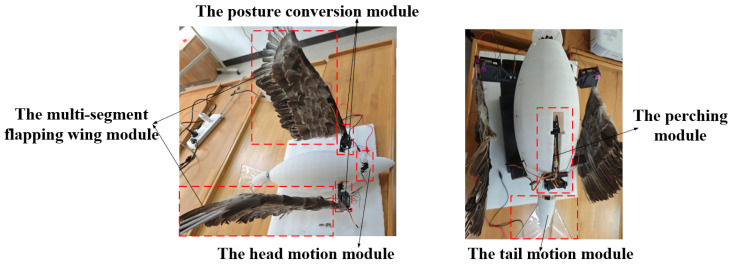
The prototype of the flapping wing aircraft with perching function.

Based on the above design method and additive manufacturing technology, we constructed a prototype of the flapping-wing aircraft with perching functionality. The prototype was processed using nylon reinforced with carbon fiber composite materials, and its feathers, arranged similarly to those of large birds such as eagles, were made using duck feathers. The prototype’s overall shape and main functional modules are depicted in Figure 18, below.

**Figure 19:**
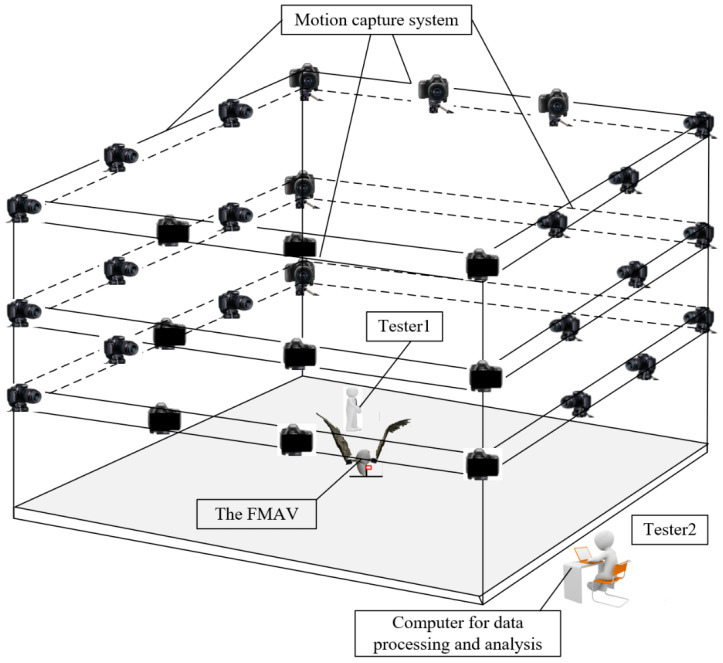

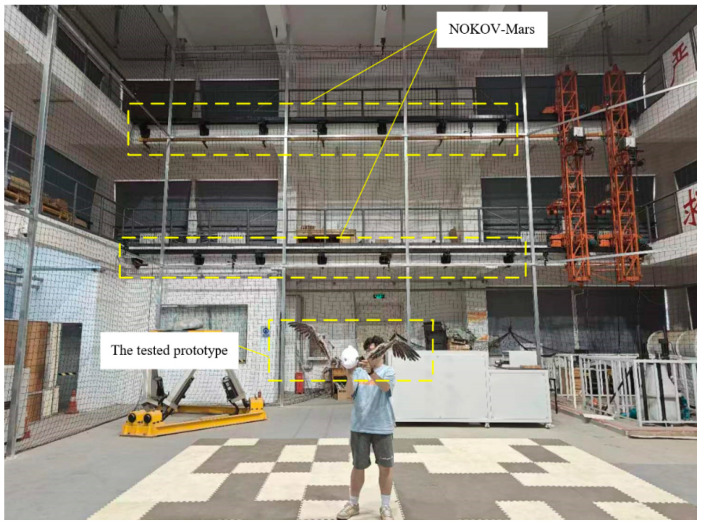
The scenario for the experiment.

To capture key parameters of the flapping-wing aircraft with perching functionality during its operation for basic flight and multi-modal movements, the prototype was placed within a 5 m × 5 m × 15 m test space. Surrounding the prototype, a motion capture system equipped with 32 infrared cameras (NOKOV-Mars) was set up. Infrared tracker data were wirelessly transmitted to a computer for data processing and analysis. The experiment scenario is illustrated in Figure 19, below.

**Figure 20:**
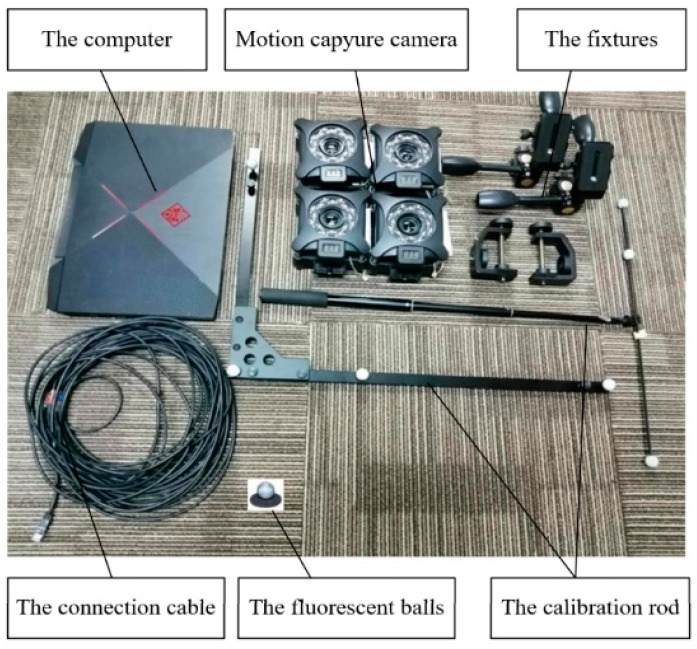
The motion capture device for the experiment.

To ensure the accuracy of the experimental measurements and avoid interference from factors such as motor performance and material stiffness, which directly affect the flapping motion parameters, the attitude transformation mechanism was manually operated, ensuring that the mechanism completes flapping, folding, and bending motions under controlled conditions, thereby securing precise and reliable experimental results. The motion capture device is shown in Figure 20, below.

**Figure 21:**
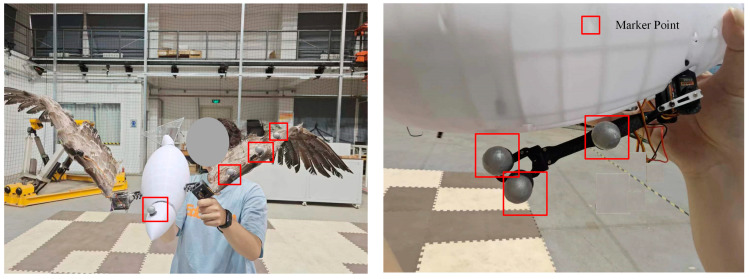
The installation locations for the fluorescent balls.

To reflect key spatial points during the motion of the prototype, fluorescent balls were installed at the following critical locations: along the leading edge of the wing, one at the wing center, one at the transition between the first and second wing segments, and one at the center of the second wing segment, to measure the motion parameters of the posture conversion module and the multi-segment flapping wing module; on the upper and lower claw of the perching module to measure the opening and closing angle of the perching module; and on the head of the flapping-wing aircraft to measure the head’s yaw and patch angles. The positions of the fluorescent balls are shown in Figure 21. The motion parameters were measured using the Xingying 1.4.0.× software developed by Nokov, which provides a distance accuracy of 1 mm and an angular measurement error of ±1°.

The flapping, folding, bending, opening and closing, and yaw and patch angles were measured three times each throughout our experiments, and the average of the three measurements for each angle was taken as the final experimental value. The calculated theoretical values, simulation values, and experimental results and comparisons are shown in Table 3.

From Table 1, it can be observed that the theoretical calculation of the flapping angle was 120.57° (60.285° for each upstroke and downstroke); compared to the Adams simulation value of 119.4° (59.7° for each upstroke and downstroke), the theoretical calculation deviation was 0.9% (deviation 1); compared to the experimental value of 118° (59° for each upstroke and downstroke), the theoretical calculation deviation was 2.1% (deviation 2). The theoretical calculation of the folding angle was 105.36°, with deviations of 1.2% compared to the simulation value, and of 1.6% compared to the experimental value. The theoretical calculation of the bending angle was 50.3°, with deviations of 1.2% compared to the simulation value, and of 5.4% compared to the experimental value. The theoretical calculation of the opening and closing angle was 120.77°, with deviations of 6.8% compared to the simulation value, and 5.2% compared to the experimental value. The theoretical calculation of the pitch angle was 20°, with a deviation of 3.7% compared to the simulation value, and 5.0% compared to the experimental value. The theoretical calculation of the yaw angle was 40°, with a deviation of 0 compared to the simulation value, and 2.5% compared to the experimental value.

Due to the manual control method employed during the experiments, the angular velocity could be adjusted in real time, ensuring that the experimental measurement values were primarily influenced by the constraints and dimensions of the attitude transformation mechanism’s components, thus avoiding angular deviations caused by material flexibility and minimizing errors from increased flapping resistance due to artificial feathers affecting motor torque. As a result, the maximum error in experimental values was 5.4%, indicating a high level of measurement accuracy. The simulation and experimental results validate the correctness of the kinematic model established in this study.

### 5.3. Prototype Performance Experiment

**Figure 22:**
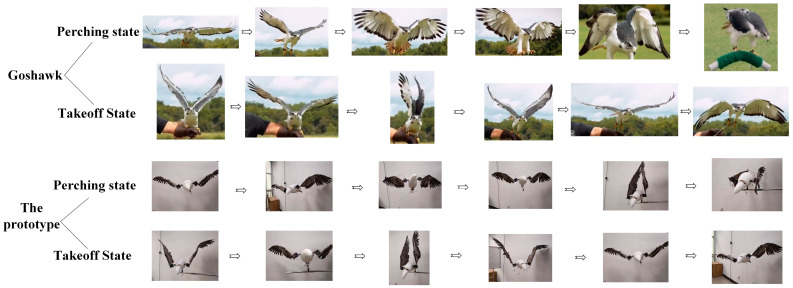
Comparison of the key postures of the flapping-wing aircraft and the corresponding hawk perching postures.

To test the basic actions required for the perching function of the flapping-wing aircraft, we conducted experiments on the two processes of perching and takeoff. A total of 12 actions were tested, and a comparison of the key postures of the flapping-wing aircraft with the corresponding hawk perching postures is shown in Figure 22, below.

The experimental results indicate that the highly biomimetic flapping-wing aircraft is capable of performing each stage of the perching process with the coordination of motors and servos. The perching module can transition the legs from a retracted to an extended position, and the claws from closed to open, during landing or perching. The posture conversion mechanism allows for multi-degree-of-freedom movement. Additionally, the head and tail can achieve pitch, yaw, and biaxial coupled motions.

**Figure 23:**
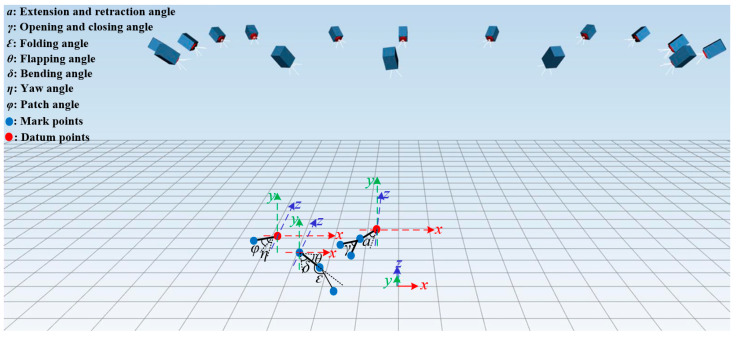
Schematic diagram of key motion parameters during perching and takeoff processes.

**Figure 24:**
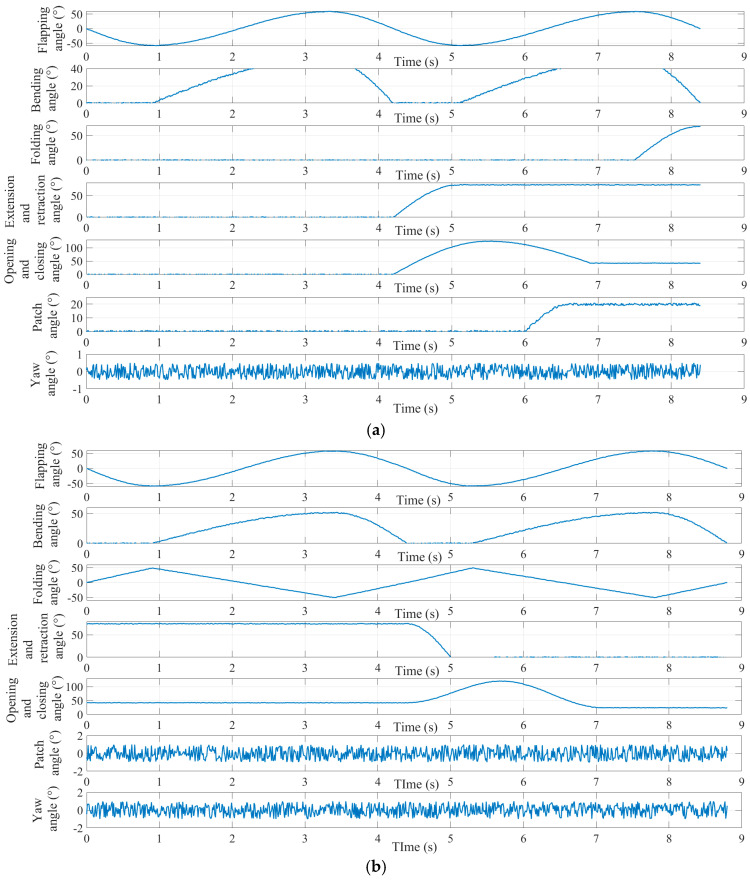
Time-varying curves of various motion parameters during the process of perching (**a**) and takeoff (**b**).

The main motion parameters during the perching and takeoff processes of the flapping-wing aircraft were measured; the definitions of the main motion parameters in the motion capture system are shown in Figure 23, and the time-varying curves of each motion parameter during the landing and takeoff processes are shown in Figure 24.

As shown in Figure 24, the perching process of the flapping-wing aircraft takes a total of 8.4 s. A single flap cycle takes 4.2 s; the flap begins at 0°, and in the first 0.9 s, the downward stroke phase occurs, reaching a maximum angle of 57.6°; then, from 0.9 s to 3.3 s, the upward stroke phase takes place, with a maximum upward angle of 58.8°; finally, from 3.3 s to 4.2 s, the second downward stroke phase follows. The wing motion throughout the flap cycle exhibits a sinusoidal-like pattern. During the upward stroke phase, the wing folding motion also occurs, with the maximum folding angle reaching 50.8°. The folding angle gradually increases during the second flap cycle, starting at 7.5 s, reaching 68.5° as the wings are drawn forward, corresponding to the forward thrusting motion of the wings as seen in Figure 22.

From 4.2 s onward, the claw extension angle increases from 0° to 75° and remains unchanged, indicating the initiation of the perching action from the second flap cycle, and corresponding to the downward extension of the hawk’s legs before landing, as shown in Figure 22. The opening and closing angle of the claw increases from 0° to 124.6° between 4.2 s and 5.5 s, then decreases to 42° by 6.9 s and remains constant, corresponding to the hawk’s claws opening during perching and then closing to grasp the perch, as seen in Figure 22. Finally, from 6 s onwards, the head pitch angle increases from 0° to 19.5° and remains unchanged, indicating the head adjustment for stability after the wings are folded during perching, as shown in Figure 22.

The takeoff process of the flapping-wing aircraft takes a total of 8.8 s. A single flap cycle takes 4.4 s, beginning at 0°, and, in the first 0.9 s, the downward stroke phase occurs, reaching a maximum angle of 58.2°; then, from 0.9 s to 3.3 s, the upward stroke phase follows, with a maximum upward angle of 57.7°; finally, from 3.3 s to 4.2 s, the second downward stroke phase follows. The wing motion throughout the flap cycle exhibits a sinusoidal-like pattern. During the upward stroke phase, the wing folding motion also occurs, with the maximum folding angle reaching 51.4°. The folding angle is positive during the downward stroke phase, with the wing moving downward; during the upward stroke phase, the folding angle becomes negative, indicating the wing’s upward motion; this coupled motion of flapping and folding is shown in Figure 22.

From 4.4 s onward, the claw stretching angle gradually decreases, from 75° to 0°, within 1.2 s, indicating that the perching action starts to reverse and the claws retract during takeoff, which corresponds to the hawk’s legs retracting after detaching from the perch, as shown in Figure 22. The opening and closing angle of the claw increases from 42° to 121.4° between 4.4 s and 5.7 s, then decreases to 24° by 7.1 s and remains constant, corresponding to the hawk’s claws opening after taking off and then closing as it prepares to stabilize during flight, as shown in Figure 22.

## 6. Discussion

We based this study on the principles of biomimicry, utilizing mechanical structures to simulate the posture of a hawk’s body parts during the perching process, and designing a highly biomimetic flapping-wing aircraft with perching capabilities. As shown in the test results in Figure 22, the flapping-wing aircraft designed in this research can achieve key postures at various stages of the perching process, mimicking those of hawks. The results in Table 3 also show that all motion parameters met the design requirements.

From the curves of the main motion parameters during the perching and takeoff processes of the flapping-wing aircraft, shown in Figure 24, it can be seen that, while the aircraft can perform the key actions at each stage of the perching process, several issues are also evident, mainly in the following two aspects:

(1) The duration of a single flap cycle is too long. The duration of a single flap cycle for our prototype is around 4 s, which is too long. Studies have shown that a mid- to large-sized flapping-wing aircraft with a mass of 500 g requires a flapping frequency of 4 Hz to achieve flight [9,28].

(2) The response time of the claws is too low. The response time for leg movement in our prototype took too long, with the leg extension taking 1.2 s and the claw opening/closing taking 1.3 s. Examples in the literature have pointed out that, for an aircraft at a flight speed of 2.5 m/s, a rapid lock onto the perch within 25 ms is necessary for the maneuverability and response speed required to meet its perching needs [21].

Clearly, the current motion parameters of the flapping-wing aircraft do not meet the requirements for normal flight and perching maneuverability or response speed, an issue that is directly related to the motor torque. Mid- to large-sized flapping-wing movements require high-torque motors/servos, but high-torque motors/servos typically require larger installation spaces. Therefore, selecting motors/servos that meet the requirements within the limited space is a key challenge in the design of flapping-wing aircraft. In the future, the two following approaches will be attempted to address the issue of insufficient torque:

(1) Replace the motors with higher torque motors.

(2) Redesign the gearbox to increase torque based on the existing motors.

Additionally, the simulation of the eagle’s flight actions at each stage, as discussed in this paper, is mainly based on the capture of movement frames from flight videos of eagles (Figure 2 and Figure 3). However, birds such as eagles can adaptively adjust their optimal posture based on real-time airflow [29]; therefore, the characterization of key postures illustrated in this paper is not comprehensive. Future research will focus on the multi-degree-of-freedom flapping-wing aircraft with perching capabilities designed in this study, specifically the impact of wing–leg coordination on the aerodynamic performance of the flapping-wing aircraft during modal transition phases, which will provide a more accurate representation of the optimal postures at various stages of multi-modal motion under different environmental conditions, offering data support and methodological guidance for the design of flapping-wing aircraft.

In bionic design, particularly in the context of the flapping-wing aircraft, the interaction between wing posture and leg/claw movements is often inspired by how real birds and other flying animals coordinate their limbs for efficient flight and landing. Research shows that wing posture primarily influences the movement of the legs and claws by altering the center of gravity and aerodynamic force distribution. The movements of the legs and claws, in turn, affect the wing posture by adapting to the required flight speed and the position of the body relative to the perching object [30,31,32,33].

The flapping-wing aircraft we designed employs a distributed drive system to achieve the movement of each functional module (Figure 4); in other words, the main actions performed by the flapping-wing aircraft (such as twisting, swinging, folding, flapping, leg extension and retraction, and claw opening and closing) are driven via one or more motors individually. However, we must acknowledge that, at this stage, we have not yet developed a coordinated control strategy for the wings, legs, and claws based on center-of-gravity adaptive adjustment or optimal aerodynamic efficiency as the objective function; nonetheless, we have laid the foundation for this research, as we have designed a flapping-wing aircraft capable of achieving the required postures during the perching and takeoff phases, an aspect that will be a focus of future research.

## 7. Conclusions

In this study, we use mechanical structures to simulate the postures of various parts of the hawk during perching, designing a highly biomimetic flapping-wing aircraft with perching functionality based on bionic principles, the main conclusions from which are as follows:

(1) Utilizing a multi-motor solution, the posture conversion mechanism, designed with a hemispherical gear structure, enables the aircraft to perform flapping, twisting, and swinging motions, as well as their coupled movements, while also allowing the wing to transition between folded and unfolded states. The perching module, designed using the screw motion principle, can open and close the claws. The head and tail motion modules, designed based on the dual-motor coupling motion principle with a limit structure, achieve pitch, yaw, and coupled motion.

(2) The deviation between the calculation and the experimental value of the flapping angle is 2.1%; specifically, the deviation for the folding angle is 1.6%, that for the bending angle is 5.4%, that for the opening and closing angle of the perching module is 5.2%, that for the yaw angle of the head and tail is 2.5%, and that for the pitch angle of the head and tail is 5%. The calculated and the experimental values are basically consistent, indicating the accuracy of the kinematic model.

The highly biomimetic flapping-wing aircraft designed in this study can replicate the perching postures of a hawk at various stages using motors, providing a theoretical foundation and testing platform for future research on dynamic modeling and control methods.

## Figures and Tables

**Table 1:** Parameters of main component.

Motion Angle	Component	Parameters (m)	Motion Angle	Component	Parameters (m)
Flapping angle	OP	0.247	Opening and closing angle	*Ox*_0_	0.0976
	PQ	0.547		*Oy*_0_	0.0793
	QR	0.3		*Ox*_1_	0.018
	OR	0.6		*Oy*_1_	0.078
Folding angle	JK	0.2		*r*	0.0294
	KL	0.385	Yaw angle	*R*_0_	0.133
	LH	0.272		*l*_0_	0.02
	JH	0.5		*l*_AB_	0.15
Bending angle	AB	0.171			
	BC	0.331			
	CD	0.445			
	AD	0.5			

**Table 2:** The comparison between the simulation results and theoretical calculations.

Parameters	Calculation Value (°)	Simulation Value (°)	Deviation (%)
Flapping angle	120.57	119.4	0.9
Folding angle	105.36	104.06	1.2
Bending angle	50.3	50.914	1.2
Opening and closing angle	120.77	128.96	6.8
Yaw angle of the head coupling motion module	40	40	0
Patch angle of the head coupling motion module	20	20.74	3.7

**Table 3:** Comparison of motion parameters results.

Parameters	Calculation Value (°)	Simulation Value (°)	Experimental Value (°)	Deviation 1 (%)	Deviation 2 (%)
Flapping angle	120.57	119.4	118	0.9	2.1
Folding angle	105.36	104.06	107	1.2	1.6
Bending angle	50.3	50.914	53	1.2	5.4
Opening and closing angle	120.77	128.96	127	6.8	5.2
Patch angle of the head coupling motion module	20	20.74	21	3.7	5.0
Yaw angle of the head coupling motion module	40	40	41	0	2.5

## Data Availability

The data for this paper come from simulation settings and measurements during the experiment, please contact the corresponding author’s email if necessary.

## References

[1] Vogel H.F., McCarron V.E.A., Zocche J.J. (2018). Use of artificial perches by birds in ecological restoration areas of the Cerrado and Atlantic Forest biomes in Brazil. Neotrop. Biol. Conserv..

[2] Escrivà A.C., López-Iborra G.M., Cortina J., Tormo J. (2019). The use of branch piles to assist in the restoration of degraded semiarid steppes. Restor. Ecol..

[3] Huang Z.F., Li S., Jiang J.G., Wu Y., Yang L., Zhang Y. (2021). Biomimetic Flip-and-Flap Strategy of Flying Objects for Perching on Inclined Surfaces. IEEE Robot. Autom. Lett..

[4] Zheng P., Xiao F., Nguyen P.H., Farinha A., Kovac M. (2023). Metamorphic aerial robot capable of mid-air shape morphing for rapid perching. Sci. Rep..

[5] Roderick W., Cutkosky M., Lentink D. (2017). Touchdown to take-off: At the interface of flight and surface locomotion. Interface Focus.

[6] De Croon G.C.H.E., Groen M.A., Wagter C.D., Remes B., Ruijsink R., van Oudheusden B.W. (2012). Design, aerodynamics and autonomy of the DelFly. Bioinspir. Biomim..

[7] Tu Z., Hui C., Liu L.M., Zhou Y.M., Romano D.R., Deng X.Y. (2021). Crawl and fly: A bio-inspired robot utilizing unified actuation for hybrid aerial-terrestrial locomotion. IEEE Robot. Autom. Lett..

[8] Ang H.S. (2012). Introduction to Micro Aircraft Design.

[9] Wang J.X., Xuan J.L., Yang X.J., Zheng R. (2024). Research progress of flapping wing aircraft with multimodal motion ability. Acta Aeronaut. Astronaut. Sin..

[10] Desbiens A.L., Cutkosky M.R. (2010). Landing and perching on vertical surfaces with microspines for small unmanned air vehicles. J. Intell. Robot. Syst..

[11] Mehanovic D., Bass J., Courteau T., Rancourt D., Desbiens A.L. (2017). Autonomous thrust-assisted perching of a fixed-wing uav on vertical surfaces. Biomim. Biohybrid Syst..

[12] Kirchgeorg S., Mintchev S. (2021). HEDGEHOG: Drone perching on tree branches with high-friction origami spines. IEEE Robot. Autom. Lett..

[13] Daler L., Klaptocz A., Briod A., Sitti M., Floreano D. A perching mechanism for flying robots using a fibre-based adhesive. Proceedings of the 2013 IEEE International Conference on Robotics and Automation.

[14] Doyle C.E., Bird J.J., Isom T.A., Kallman J.C., Bareiss D.F., Dunlop D.J., King R.J., Abbott J.J., Minor M.A. (2012). An avian-inspired passive mechanism for quadrotor perching. IEEE/ASME Trans. Mechatron..

[15] Broers K.C.V., Armanini S.F. (2022). Design and testing of a bioinspired lightweight perching mechanism for flapping-wing MAVs using soft grippers. IEEE Robot. Autom. Lett..

[16] Zhang H.J., Lerner E., Cheng B., Zhao J.G. (2021). Compliant bistable grippers enable passive perching for micro aerial vehicles. IEEE/ASME Trans. Mechatron..

[17] Roderick W.R.T., Cutkosky M.R., Lentink D. (2021). Bird-inspired dynamic grasping and perching in arboreal environments. Sci. Robot..

[18] Graule M.A., Chirarattananon P., Fuller S.B., Fuller S.B., Jafferis N.T., Ma K.Y., Kornbluh M., Wood R.J. (2016). Perching and takeoff of a robotic insect on overhangs using switchable electrostatic adhesion. Science.

[19] Chirarattananon P., Ma K.Y., Wood R.J. (2016). Perching with a robotic insect using adaptive tracking control and iterative learning control. Int. J. Robot. Res..

[20] Zhao L., Wang H., Jiang W., Jiao Z., Zheng Y. (2020). Small Aircraft Climbing Device Level Small Aircraft. https://www.patent9.com/patent/202011326291.5.html.

[21] Zufferey R., Tormo-Barbero J., Feliu-Talegón D., Nekoo S.R., Acosta J.A., Ollero A. (2022). How ornithopters can perch autonomously on a branch. Nat. Commun..

[22] Feliu-Talegon D., Acosta J.A., Ollero A. (2021). Control aware of limitations of manipulators with claw for aerial robots imitating bird’s skeleton. IEEE Robot. Autom. Lett..

[23] Maldonado F.J., Acosta J.Á., Tormo-Barbero J., Grau P., Guzmán M.M., Ollero A. Adaptive nonlinear control for perching of a bioinspired ornithopter. Proceedings of the 2020 IEEE/RSJ International Conference on Intelligent Robots and Systems (IROS).

[24] Feliu-Talegon D., Acosta J.Á., Suarez A., Ollero A. (2020). A bio-inspired manipulator with claw prototype for winged aerial robots: Benchmark for design and control. Appl. Sci..

[25] Zbikowski R., Galinski C., Pedersen C.B. (2005). A four-bar linkage mechanism for insect-like flapping wings in hover; concept and an outline of its realization. ASME J. Mech. Des..

[26] Zhu B., Ang H.S., Guo L. (2007). Design and analysis of new 3D insect-like flapping-wing mechanism. Trans. Nanjing Univ. Aeronaut. Astronaut..

[27] Ji B., Zhu Q.L., Guo S.J., Yang F., Li Y.S., Zhu Z.G., Chen S., Song R., Li Y.B. (2020). Design and experiment of a bionic flapping wing mechanism with flapping–twist–swing motion based on a single rotation. AIP Adv..

[28] Shyy W., Shyy M., Ljungqvist D. (1999). Flapping and flexible wings for biological and micro air vehicles. Progress. Aerosp. Sci..

[29] Ma D.F., Song B.F., Gao S.J., Xue D., Xuan J.L. (2024). Designing efficient bird-like flapping-wing aerial vehicles: Insights from aviation perspective. Bioinspir Biomim..

[30] Altshuler D.L., Bahlman J.W., Dakin R., Gaede A.H., Goller B., Lentink D., Segre P.S., Skandalis D.A. (2015). The biophysics of bird flight: Functional relationships integrate aerodynamics, morphology, kinematics, muscles, and sensors. Can. J. Zool..

[31] Liu H., Ravi S., Kolomenskiy D., Tanaka H. (2016). Biomechanics and biomimetics in insect-inspired flight systems. Philos. Trans. R. Soc. B Biol. Sci..

[32] Chen Z., Xie Y., Meng X. (2024). Unsteady aerodynamic forces of tandem flapping wings with different forewing kinematics. Biomimetics.

[33] Han J.J., Hui Z., Tian F.B., Chen Z. (2021). Review on bio-inspired flight systems and bionic aerodynamics. Chin. J. Aeronaut..

